# Lipotoxic Proximal Tubular Injury: A Primary Event in Diabetic Kidney Disease

**DOI:** 10.3389/fmed.2021.751529

**Published:** 2021-10-25

**Authors:** Hua Wang, Shu Zhang, Jia Guo

**Affiliations:** ^1^Department of Pharmacy, The First Affiliated Hospital of Zhengzhou University, Zhengzhou, China; ^2^Department of Nephrology, The First Affiliated Hospital of Zhengzhou University, Zhengzhou, China; ^3^Department of Nephrology, Nephropathy Research Institutes of Zhengzhou University, Zhengzhou, China

**Keywords:** lipotoxicity, tubular injury, diabetic kidney disease, primary event, lipid accumulation

## Abstract

The pathogenesis of diabetic nephropathy is a complex process that has a great relationship with lipotoxicity. Since the concept of “nephrotoxicity” was proposed, many studies have confirmed that lipotoxicity plays a significant role in the progression of diabetic nephropathy and causes various renal dysfunction. This review will make a brief summary of renal injury caused by lipotoxicity that occurs primarily and predominantly in renal tubules during diabetic progression, further leading to glomerular dysfunction. The latest research suggests that lipotoxicity-mediated tubular injury may be a major event in diabetic nephropathy.

## Introduction

Diabetic kidney disease (DKD) is a common complication of diabetes mellitus (DM) and a leading cause of renal failure. Approximately 30–40% of patients with T1DM and T2DM develop DKD, and approximately 50% of them can progress to end-stage renal disease (ESRD) (1). Currently, the prevalence, mortality, and cost of DKD are high (2). According to the 2018 US Renal Data System report, the prevalence of end-stage renal disease due to diabetes continues to increase and is expected to be 44% by 2030 (3). However, understanding of DKD is still insufficient and the effective prevention and treatment rate are poor. In clinical practice, DKD is diagnosed by proteinuria, decreased estimated glomerular filtration rate (GFR), or both (4). However, the precision and prognostic value of these biomarkers in the early stages of the disease are limited, so there is urgent need to find new indicators for the early diagnosis of DKD.

Lipid accumulation is a common phenomenon in patients with DKD (5). Since Moorhead first proposed the “hypothesis of renal toxicity” in 1982 (6), increasing evidence supports the hypothesis that lipotoxicity leads to renal tubular epithelial cell injury and promotes renal disease progression. Lipotoxicity has been found to cause a series of renal injuries, including mitochondrial dysfunction, tubular epithelial cell apoptosis, tubular atrophy, and tubulointerstitial fibrosis. Interestingly, injury occurs preferentially in renal tubules, unlike the traditional concept of diagnosis and treatment of DKD focusing on the glomeruli, and this may provide a new direction for future research in DKD. In this review, we will summarize the early renal injury caused by lipotoxicity and discuss whether tubular lipotoxicity can be used as an indicator for early prediction of DKD.

## Lipid Metabolism in the Renal Tubule

**Figure 1:**
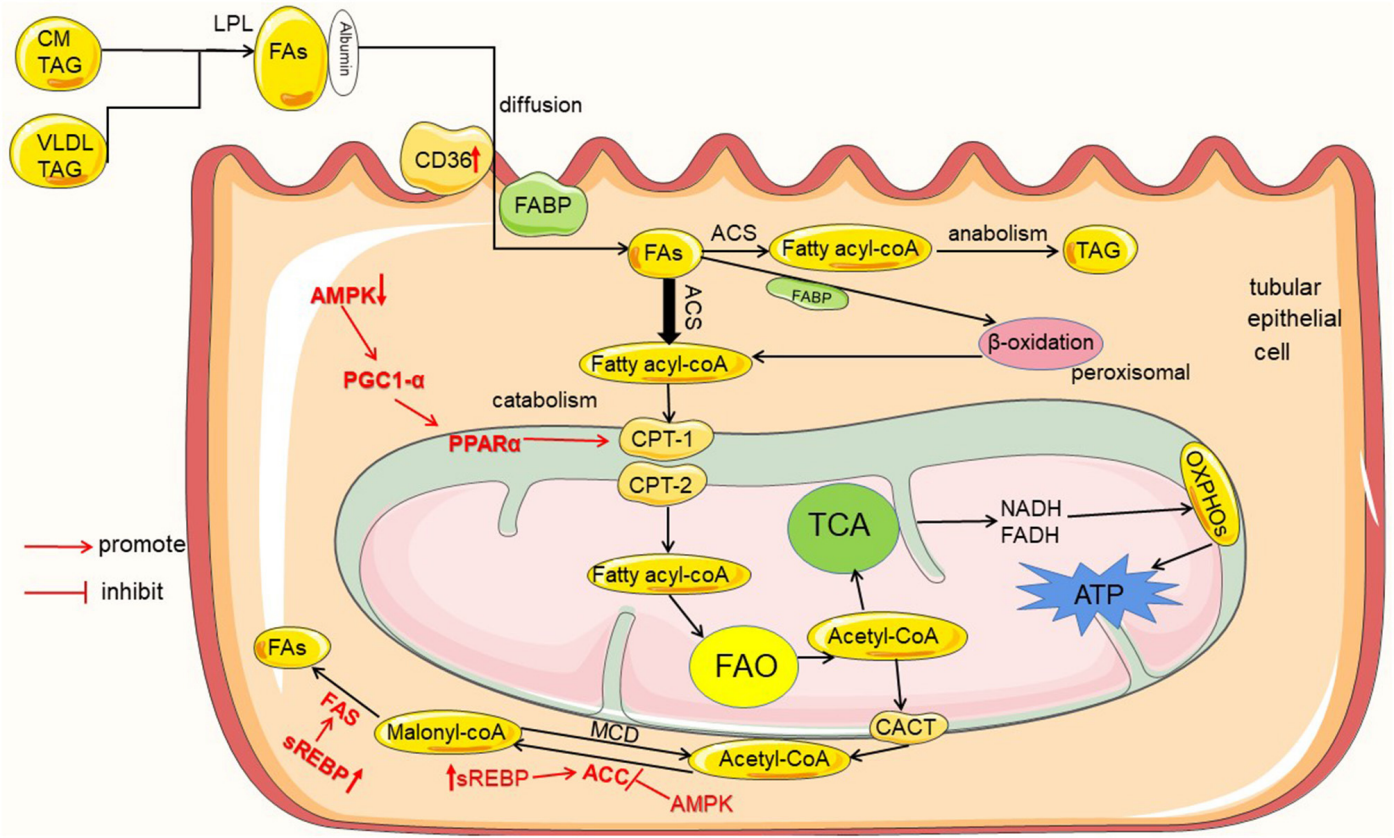
Lipid metabolism in renal tubules and the regulation mechanism in diabetes mellitus. Tubules mainly utilize the metabolism of FAs as an energy source, and the metabolism of FAs includes catabolism and anabolism. FAs entering the renal tubules mainly come from the blood circulation. Initially, FAs are esterified in the form of VLDL and CM-TAG; then, esterified FAs are catabolized by LPL to release FAs. Extracellular FAs enter the cell by autonomous diffusion or transporters as CD36 and FABP. FAs into the cell are activated by ACS to fatty acyl-CoA, and then part of fatty acyl-CoA enters the mitochondria via CPT-1 and CPT-2 for catabolism to produce acetyl-CoA, which enters TCA to produce ATP required by the kidney; and excess fatty acyl-CoA enters the anabolic pathway to generate TAG. In addition, the product of FAO acetyl-CoA can also be transported out of mitochondria by CACT and converted to malonyl-CoA by ACC, and malonyl-CoA resynthesizes new FAs by FAS. In diabetes and high glucose conditions, the proteins are high- or down regulated in the uptake, metabolism and synthesis of FAs, which are marked in red color.

The kidney is one of the most energy-demanding organs in the human body, and numerous studies have shown that the kidney mainly uses fatty acid oxidation (FAO) as its energy source (7). Because fatty acids (FAs) metabolism produce 3 times more adenosine triphosphate (ATP) than glucose (8). Most renal tubular epithelial cells (TECs) have low metabolic flexibility toward glycolysis and rely on FAs as energy source at baseline (9). This was shown *in vivo* studies measuring ATP synthesis by tracking isotope-labeled FAs with NMR in rat kidney, which indicated that FAs are a preferred fuel (6). Renal FAO occurs mainly in mitochondria, and tubular cells contain a large number of mitochondria (10). For example, the human proximal convoluted tubules contain abundant large mitochondria, which occupy about 16.3% cell volume (7). Thus, renal tubules are the core site of renal energy metabolism (10). In blood, more than 90 % of FAs are esterified and circulate as triglyceride (TAG) within very low-density lipoprotein (VLDL) and chylomicron particles (CM). Esterified FAs are initially catabolized by lipoprotein lipase (LPL) to release non-esterified fatty acids (NEFAs) (11). Then, NEFAs enter the cells with the help of fatty acid transporters and to be metabolized (as shown in Figure 1).

### Renal Tubular FAs Uptake

The first step in FAs metabolism is the uptake of extracellular FAs, and this involves the participation of a variety of fatty acid transporters, such as cluster of differentiation 36 (CD36) (12) and fatty acid binding proteins (FABPs) (13). Some can also enter cells by simple diffusion.

CD36, also known as scavenger receptor B2, is a membrane protein that is widely expressed (12). In the kidney, CD36 is highly expressed in the epithelial cells of the proximal tubules and distal tubules (14). CD36 mediates the binding and intracellular uptake of long-chain fatty acids (LCFAs), oxidized lipids and phospholipids (ox-LDL), advanced oxidative protein products, thrombospondin, and advanced glycation products (15). FABPs are a family of highly expressed intracellular proteins, with 15 members currently found, and FABP1 is found to be expressed in proximal tubular epithelial cells (16–18). The functions of FABP1 include: facilitating the uptake of intracellular LCFA; transporting LCFA to peroxisomes for beta oxidation; transporting LCFA and long-chain fatty acid acyl-CoA (LCFA-CoA) to mitochondria for oxidation (18).

### Beta-Oxidation of FAs

FAs are transported into cells after binding to transporters. FAs that enter the cell are activated by acyl-CoA synthetase (ACS) to fatty acid acyl-CoA (fatty acyl-CoA) (13). Fatty acyl-CoA is transported into the mitochondrial matrix via carnitine shuttles (CPT1, CPT2, CACT) (11). In the mitochondrial matrix, fatty acyl-CoA are degraded *via* β-oxidation, a cyclic process consisting of four enzymatic steps, produces acetyl-CoA (19). Then, acetyl-CoA enters the tricarboxylic acid cycle (TCA) to generate FADH2 and NADH. Finally, ATP is produced by oxidative phosphorylation (OXPHOS) (20). Excess acetyl-CoA can also be transported out of the mitochondria by carnitine acetyltransferase (CACT), which in turn synthesizes new FAs (21). On the other hand, very long chain fatty acids (VLFAs) are initially oxidized in the peroxisome, releasing acetyl CoA until their chain length is shortened to eight carbons and then transported to the mitochondria to complete oxidation (13).

### Synthesis of Fatty Acids and Triglycerides

Metabolism of FAs includes catabolism and anabolism. In tubular cells, some of fatty acyl-CoA enters the mitochondria for catabolism and produces the energy required by the kidney; excess fatty acyl-CoA then enters the anabolic pathway and generates TAG for storage. In addition, acetyl-CoA generated by FAs through beta-oxidation in the mitochondria can also be transported out by CACT (21). It is then converted to malonyl-CoA by acetyl-CoA carboxylase (ACC), which re-synthesizes new fatty acids (22, 23).

The intermediates or enzymes related to fatty acid metabolism are regulated by some enzymes or transcription factors, such as AMP protein AMPK), peroxisome proliferator-activated receptor α (PPARα), peroxisome proliferator-activated receptor γ coactivator-1α (PGC-1α), sterol regulatory element binding proteins (SREBP), and carbohydrate response element binding protein (ChREBP). AMPK is an enzyme that plays a key role in cellular energy homeostasis. AMPK can inhibit ACC activity, hence reducing malonyl-CoA levels, and increase CPT1 activity, thus promoting FAO (9). It is now also found that AMPK is able to activate PPARα to stimulate fatty acid oxidation by increasing PGC-1α activity (24). In addition, SREBP and ChREBP can promote the expression of ACC and FAS, thereby increasing fatty acid synthesis (22, 23).

## Tubular Lipid Metabolism in Diabetes Mellitus

Chronic kidney disease is associated with altered lipid metabolism and lipid accumulation (25). DKD is a major cause of chronic kidney disease (CKD), and lipid accumulation is a common phenomenon in patients with DKD (5). Metabolic changes associated with diabetes are reported to contribute to early DKD (26), and abnormal metabolism is associated with DM Type 1 or type 2 (27). Under diabetic or continuous high glucose conditions, renal tubular lipid metabolic disorders and lipid accumulation are mainly related to the imbalance between the uptake, metabolism and synthesis of FAs (as shown in Figure 1).

### Increased FAs Uptake by the Tubules

In tubular cells, the proteins associated with FAs uptake are mainly CD36 and FABPs (15, 18). In the early stages of diabetes, increased levels of CD36 are clearly observed (28). TECs-specific overexpression of CD36 transgenic mice showed an increase of lipid accumulation in TECs (29). Thus, increased expression of CD36 leads to increased uptake of intracellular FAs, and excessive deposition of FAs causes accumulation of tubular lipids.

### Decreased FAs Beta-Oxidation

PPARα and PGC-1α are key transcription factors for protein (22)expression of CPT-1 (30), which is the rate-limiting enzyme for fatty acid acyl-CoA into mitochondria (23). Under hyperglycemic conditions, it has been demonstrated that down-regulation of FAO genes such as PPARα, PGC-1, and CPT-1 leads to decrease in FAs β-oxidation (31–33). In addition, hyperglycemia caused the reduced activity of AMPK (23), and the expression of downstream of the AMPK pathway PPARα, PGC-1, and CPT-1, is also decreased. Eventually the oxidation of FAs is impaired, causing the accumulation of intracellular lipids.

### Increased Synthesis of FAs and Accumulation of TAG

On the one hand, under high glucose conditions, the expression of SREBP and ChREBP is activated, which promotes the expression of ACC, FAS (23) and the synthesis of FAs is increased. On the other hand, surplus FAs due to impaired β-oxidation are restored in the form of TAG inside the renal cells by activating the fatty acid synthesis pathway (23). As a result, the synthesis of FAs are increased in renal tubular cells, and fatty acid transport is impaired and/or FAO is reduced. These cause dysregulation of the metabolism of FAs, as well as excessive intracellular production of FAs and TAG. Finally, there is accumulation of lipids in kidney tubules (22).

### Formation of Lipotoxicity

**Figure 2:**
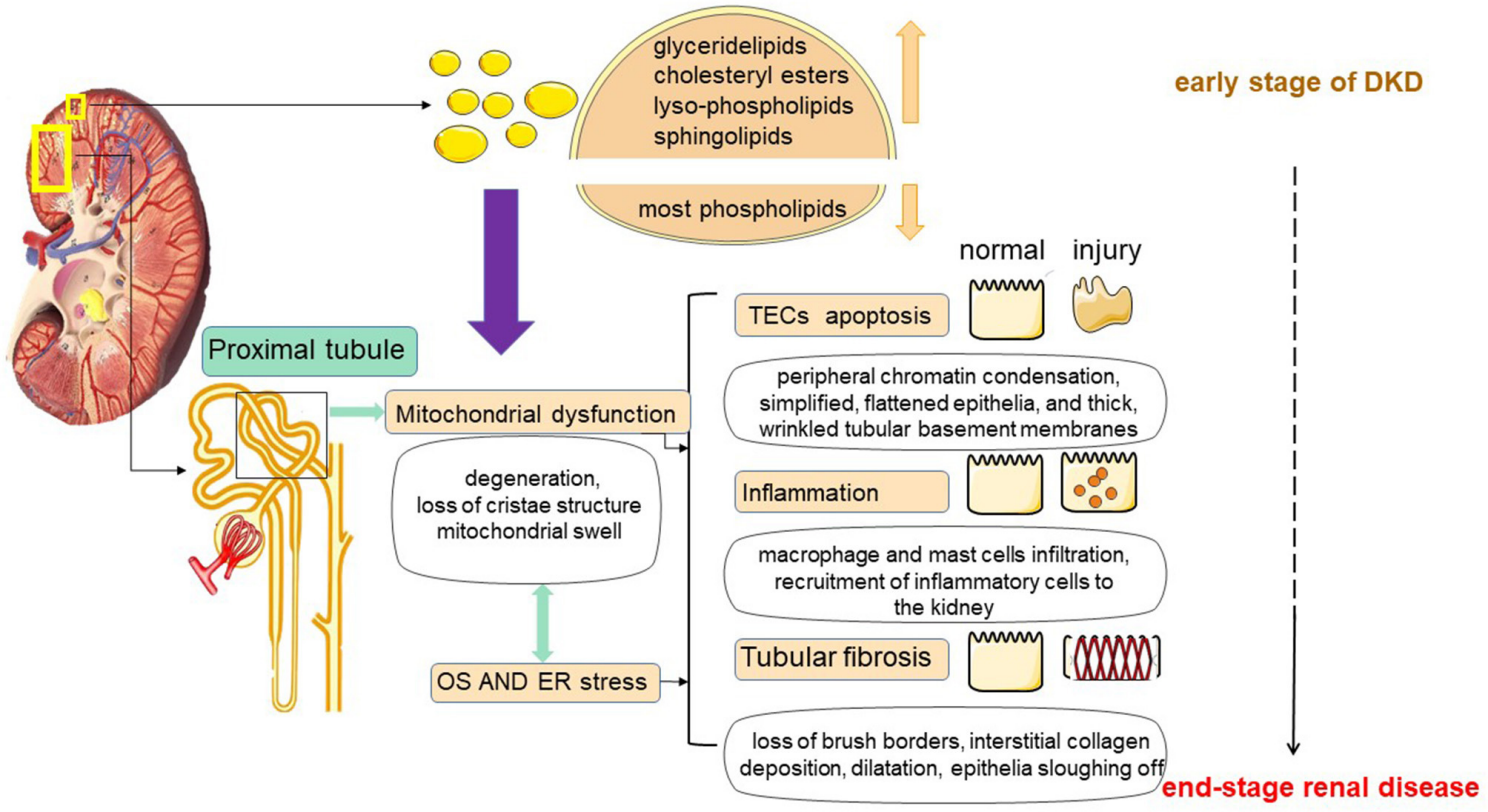
Lipotoxicity-mediated tubular injury. Tubular lipotoxicity leads to the development of a series of renal injuries including tubular epithelial cell apoptosis, inflammation and tubulointerstitial fibrosis. First, in renal tubules, lipotoxicity may cause mitochondrial dysfunction, involving massive ROS production, and inducing oxidative stress (OS) and ER stress. Second, lipotoxicity may cause OS and ER stress, which in turn may cause mitochondrial damage. At the same time, OS and ER stress may also cause damage to mitochondria. In addition, mitochondrial dysfunction, OS and ER stress together do harm to tubular cells.

In diabetes, the imbalance of fatty acid uptake, oxidation and synthesis in renal tubular cells causes the over-production of FAs, and when it exceeds the utilization rate of FAs by renal tubules, excessive FAs and TAG are formed and deposited in renal tubules. Recently a study on comprehensive lipidome profiling of the kidney cortex during early stage of DKD showed that there were distinct lipidomic signatures in the kidney. The levels of glyceride lipids, especially cholesteryl esters, lyso-phospholipids and sphingolipids, including ceramide and its derivatives, exhibited a dramatical elevation, while the levels of most phospholipids showed a decline in the DKD kidney cortex. The lipid metabolic disturbance does shed a light on the mechanism of renal dysfunction on the early stage of diabetes (26). Accumulation of large amounts of lipids causes the production of proinflammatory factors, toxic intermediate metabolites, which in turn induce an inflammatory response, oxidative stress, and ultimately trigger cell damage (34). This abnormal accumulation of lipids in non-adipose tissue leads to dysregulation of intracellular homeostasis, causing the phenomenon of cellular damage known as lipotoxicity (35) (as shown in Figure 2).

Analysis on genetic predisposition to diabetic kidney disease implicates genes involving in lipotoxicity. A single nucleotide polymorphism in a noncoding region of the acetyl-CoA carboxylase (ACACB) gene (rs2268388), which plays a critical role in FA oxidation, showed the strongest association with proteinuria in numerous cohorts of individuals from different genetic backgrounds. This genetic risk variants can induce tubular dysfunction by promoting ACACB-mediated inhibition of CPT1 and reducing FA oxidation (36). A case-control study found that the ε2 and ε3 alleles, corresponding coding proteins E2 (Arg158 → Cys), and E3 (parent isoform) of Apolipoprotein E (APOE) influenced lipid profile, and gave rise to independent risk factors of DKD in type 2 diabetes. ApoE2 has the lowest binding ability to apoE receptor, leading to impaired liver uptake and clearance of chylomicrons (CM) or VLDL remnants. Apo ε3, was closely related with significant elevation in total cholesterol and triglyceride levels in DKD patients (37).

Epigenetic factors, including long noncoding RNAs and microRNAs, may act as an important role in lipid metabolism. Farnesoid x receptor (FXR), deficiency of which mediates diabetes acceleration of nephropathy in T1DM, inhibits SREBP-2 and elevates miR-29a, thus relieving renal fibrosis. MTHFR, an enzyme in folate cycle and homocysteine metabolism, indirectly regulates lipid metabolism. *MTHFR* 1298A/C variant is closely associated with DN (38). Moreover, miRNAs may affect the effects of hypolipidemic drugs. Lovastatin reduces miR-33 family members, which in turn suppress SREBP-2 and cholesterol synthesis (39).

## Lipotoxicity-Mediated Renal Tubular Injury

In 1982, Moorhead first proposed the hypothesis of “renal toxicity” and explained that chronic progressive renal disease may be mediated by abnormal metabolism of lipids, which may contribute to the progression of renal insufficiency (6). Since 1982, more and more studies have demonstrated that abnormal lipid metabolism has a great relationship with kidney injury. Furthermore, the nephrotoxicity by lipids is not only a cause of but also a consequence of renal disease (7, 40). In DKD, renal tubular lipid accumulation is a common phenomenon, and excessive tubular ectopic fat deposition can further trigger lipotoxicity (5, 35). Tubular lipotoxicity leads to the development of a series of renal injuries such as oxidative stress (OS), endoplasmic reticulum stress (ER), tubular epithelial cell apoptosis, tubulointerstitial fibrosis (TIF), mitochondrial dysfunction and inflammation, etc. (41–43). Lipotoxicity is a mechanism of TECs injury and is associated with a progressive decline in renal function (44) (as shown in Figure 2).

### Renal Tubular Epithelial Cell Apoptosis by Lipotoxicity

Apoptosis is programmed cell death. Studies have shown that there is apoptosis in TECs in diabetes, and a variety of apoptotic pathways are related to renal tubular atrophy. TECs apoptosis may be a cause of renal tubular atrophy (41). Under persistent apoptotic TECs insults, the resulting pathology exhibited interstitial capillary rarefaction, and tubular atrophy characterized by simplified, flattened epithelia, and thick, wrinkled tubular basement membranes (41). In the presence of albumin, tubular cell apoptosis is often considered to be closely related to albumin (45). However, recent studies have shown that even under physiological conditions, nephrotic amounts of albumin can be normally reabsorbed in tubules (46), which indicate that albumin itself is not toxic to tubules. Christine Ruggiero showed that albumin bound FAs, but not albumin itself mediated apoptosis of TECs. Intracellular FAs and LC-CoA accumulate to levels that exceed the tubular cell metabolic and storage threshold, causing lipotoxicity that can lead to apoptosis (41). Tamaki Iwai also demonstrated in related studies that lipotoxicity caused by accumulation of FAs induces apoptosis in TECs (44).

Lipotoxicity causes mitochondrial damage, leading to dysregulation of tubular lipid metabolism, and massive accumulation of fatty acids induced by incomplete FAO, which promotes ROS production. ROS can further drive tubular epithelial cell apoptosis and affect normal renal function (47).

### Tubular Fibrosis by Lipotoxicity

Tubular fibrosis is a powerful predictor of chronic kidney disease progression, which is often accompanied by the phenomenon of tubular lipid accumulation (43), receiving much attention, particularly in DKD. It has been proposed that excess accumulation of triglycerides induces cellular lipotoxicity and has the potential to contribute to the development of fibrosis (5, 48). In unilateral ureteral obstruction (UUO)-induced mice, the accumulation of lipid droplets was found in the kidney on day 7 after surgery. The kidney morphology exhibited degeneration of tubular epithelia with loss of brush borders and dilatation, accompanied by interstitial collagen deposition in UUO groups. It showed tubular epithelial disruption with epithelia sloughing off and shedding of PAS-positive material in the tubular lumina. Treatment with BMS309403, a fatty acid-binding protein 4 (FABP4) inhibitor, alleviated lipid deposition of TECs, as well as interstitial fibrosis by regulating peroxisome proliferator-activated receptor γ (PPARγ) and restoring FAO-related enzyme activities, thus enhancing FAO in TECs (49). In the DKD model, TEC-specific high expression of CD36 caused lipid accumulation, and it was found that the level of triglycerides and long-chain fatty acids alone was not sufficient to induce the development of fibrosis (10). However, when FAO is deficient in PTCs, it contributes to the development of renal fibrosis. Studies suggest that mitochondrial fatty acid oxidation plays a key role in the development of renal fibrosis. Activation of ATF6α, a transcription factor of the unfolded protein response and an upstream regulator of fatty acid metabolism, inhibits PPARα expression and subsequent FAO, followed by apoptosis and further fibrosis of PTCs. Atf6α-/- mice had maintained expression of PPARα and also decreased tubular lipid accumulation, resulting in the amelioration of apoptosis; and less tubulointerstitial fibrosis with reduced expression of α-smooth muscle actin, and collagen I (43).

In addition, lipotoxicity causes mitochondrial damage and produces large amounts of ROS. ROS can induce the expression of pro-fibrogenic factors, such as transforming growth factor-beta (TGF-β) and plasminogen activator inhibitor-1 (PAI-1), and therefore also plays a role in promoting tubular fibrosis (50).

### Inflammatory Response by Lipotoxicity

Inflammatory response is an important aspect of tubular injury in DKD. An increasing number of studies have demonstrated that lipotoxicity is an important stimulus for systemic inflammation. Lung-Chih Li has found that the levels of interleukin-1β and interleukin-18 were up-regulated in both diet-fed mice and TECs treated with palmitic acid. In the same conclusion, Xianghui Chen reported palmitic acid could enhance the expression of interleukin-1β and interleukin-18. Besides, FA could increase the mRNA levels of the inflammatory markers F4/80 and MCP-1 (51). These results suggest that accumulation of lipids induces renal tubular inflammation (52). The intensity of adipose differentiation-associated protein (ADRP) and SREBP-1 was markedly upregulated and positively correlated with inflammation. It testified the potential role of ectopic accumulation in renal tubular injury and inflammation in DKD, and confirmed that excess lipids do promote an inflammatory response (53). Macrophages infiltration into the kidney, and monocyte and macrophage recruitment and the circulation cytokine release culminate in inflammatory-related morphological changes. Other cells such as mast cells also infiltrate the tubule-interstitium and releases inflammatory factors and proteolytic enzymes (54).

### The Mechanism of Renal Tubular Injury by Lipotoxicity

#### Mitochondrial Dysfunction by Lipotoxicity

Accumulating evidence suggests that mitochondrial damage and dysfunction are major causes of CKD pathogenesis (55). It has also been shown that abnormalities in tubular mitochondrial structure and dysfunction may be the earliest manifestations of renal disease. In DKD, mitochondrial fission and fragmentation occur more often in proximal tubules. The accumulation of lipid drops in mitochondrial could promote the loss of cristae structure, mitochondrial swelling and degeneration, restraining optimal energetic functioning (35). As early as 4 weeks after experimental DM induction, evidence of impaired mitochondrial ATP production and organelle fragmentation in TECs was found, and these changes preceded increased excretion of proteinuria, abnormal glomerular morphology, and even increased renal injury molecule-1 (KIM-1), suggesting that they may be primary abnormalities (56).

Lipid is not only an important energy source, but also an important part of mitochondrial membrane structure. Unbalanced lipid metabolism can hinder mitochondrial dynamics, leading to changes in mitochondrial lipids and dysfunction (42). Lipids are also substrate of FAO in mitochondria to meet the high energy demand of the kidney (7). The dysregulated metabolism provides more albumin-bound FAs transport to renal cells, leading to mitochondrial overload. Persistent elevated levels of FAs constantly activate mitochondrial dysfunction, leading to the occurrence of incomplete FAO and the production of reactive oxygen species (ROS) (23). In turn, dysregulated mitochondrial oxidation and increased production of ROS further cause impaired mitochondrial aptamer utilization, accumulation and finally renal lipotoxicity (42). Compared with healthy kidneys, the genes and enzymes involved in the renal FAO pathway in patients with DKD have been revealed down-regulation, especially some key transcriptional regulators, such as PPARα, and CPT1 (5). Therefore, lipotoxicity and mitochondrial dysfunction can fall into a vicious cycle.

#### Oxidative and ER Stress by Lipotoxicity

Oxidative stress (OS) is closely related to ROS generation as the imbalance toward an increasing oxidative environment (57). The production of ROS mainly includes two ways: intercellular ROS in mitochondrial; renal ROS promoted by NADPH oxidase (NOX). Production of ROS by lipotoxicity may cause oxidative damage to the tubule by changing renal pressure and blood pressure (50). Mitochondria are also a key target for the destructive effects of ROS. Oxidative damage leads to mitochondrial dysfunction and loss of mitochondrial membranes, triggering mitochondrial permeability transition (MPT) and/or release of proapoptotic proteins, such as cytochrome c, to induce cell death (58).

The endoplasmic reticulum (ER) is an organelle important for lipid metabolism regulation, protein synthesis, post-translational modification, and trafficking (58). Dysregulation of ER homeostasis is known as “ER stress”. Hai-Lu Zhao have demonstrated that excessive ectopic fat deposition in the kidney and lipid overload in intracellular organelles can lead to ER stress (59). In addition, some related experiments have also shown that dietary saturated fatty acids induce ER stress in the kidneys of animal models and in cells cultured from the kidneys (60, 61). ER stress is associated with many pathological conditions, such as inflammation, apoptosis and metabolic disorders (21, 61, 62).

## Renal Tubular Injury: Center for the Development of DKD

DKD as a common complication of type 1 and 2 DM, is the main cause of CKD and characterized by glomerulosclerosis, tubulointerstitial fibrosis, and renal vascular disease (63). In tradition, the primary clinical symptom of DKD is increased urinary albumin excretion (microalbuminuria: 30 mg/24 h−300 mg/24 h) (64). In addition, studies have shown that absolute ultra-physiological elevation of GFR is observed in the early stage of diabetes in 10–67% and 6–73% of patients with type 1 and type 2 DM, respectively (65), and this hyperfiltration phenomenon is associated with the development and progression of DKD (66). Therefore, in clinical practice, the earliest and obvious features of DKD are microalbuminuria and increased GFR, which are generally used as early diagnostic markers of DKD. Both of them are also considered as markers of glomerular damage, so research on the pathogenesis of DKD has been focused on glomerular injury. Although there is no doubt that glomerular injury is a major factor in DKD, there is recently increasing evidence that renal tubules underlie the early pathogenesis of DKD (67). Urinary neutral gelase-associated lipoprotein (NGAL), a marker of tubulointerstitial damage, has been showed increased expression in DM patients with normal microalbuminuria, suggesting that tubular injury may precede glomerular disease (68). In addition, some studies have suggested that tubular injury may cause abnormal glomerular function (69), implying that tubular lesions may be the center of DKD development.

### Tubular Injury Precedes Glomerular Injury

#### Microalbuminuria Due to Tubular Injury

For a long time, microalbuminuria test has been used as a standardized means of early DKD detection (10). Generally, the mechanisms underlying microalbuminuria in the early stages of DKD are attributed to increased glomerular filtration due to hyperfiltration or glomerular barrier injury, or a combination of both (70). However, Hanet reported that while urinary albumin increased, urinary concentrations of N-acetylβ-D-glucosaminidase (NAG), a marker of tubular injury, also increased (71), suggesting that urinary albumin excretion correlates well with markers of tubular dysfunction. Wagner found that tubular cells could increase absorbtion of albumin after an increase in glomerular endogenous albumin leakage. It showed that tubular cells are able to cope with acute albumin overload. These results suggest that renal tubules can regulate the albumin excretion rate (72). In addition, some researchers have proposed that proteinuria can also occur in nephrotic states with no change in glomerular permeability (73). Study on the glomerular phenotype in 15 mice with congenital nephrotic syndrome, some of them died after 5 weeks and alive mice have shown essentially no change in glomerular permeability, but over 100-fold increase in proteinuria (74). Animal nephrotoxicity studies have also shown that albuminuria is a highly sensitive marker of early tubular toxicity in the absence of glomerular pathology (75). The above results indicate that the glomerular effect on proteinuria may be indirect, and albuminuria may be mainly controlled by renal tubules (74, 76).

#### Glomerular Hyperfiltration by Tubular Injury

Despite difficulties in precise definition or thresholds, elevated GFR as a marker of glomerular hyperfiltration occurs early in the clinical course of DKD and is considered as an important factor in the development and progression of renal damage (66, 70). Increased GFR is generally thought to be due to increased intraglomerular pressure (causing barotrauma) and renal blood flow, resulting from an imbalance of vasoactive humoral factors that control tension in the pre-post-glomerular arterioles (65). But now an increasing number of studies have shown that the pathogenesis of glomerular hyperfiltration is complex and proposed that tubular function plays an important role in regulating glomerular filtration in DM (77, 78).

A study exploring the effect of glomerular hyperfiltration on tubular dysfunction reported that two markers of tubular injury, NGAL and KIM-1 were excreted in the urine of patients with glomerular hyperfiltration and positively correlated with GFR (79). The results suggest that glomerular hyperfiltration is associated with changes in tubular function in patients with DM. Besides, similar results have been found. Treatment with empagliflozin, an inhibitor of the sodium-glucose transporter (SGLT2), in T1DM patients under hyperfiltration results in reductions in GFR independent of its effect on plasma glucose levels over 8 weeks (80). The above findings all demonstrate that tubular events may dominate in diabetes.

In recent years, there have also been some views proposing the “tubular theory” of hyperfiltration to describe diabetes-related abnormalities with close interaction between glomeruli and tubules. That is enhanced tubular sodium (and glucose) reabsorption, tubular growth, and up-regulation of tubular sodium-glucose cotransporters (SGLTs) and sodium-hydrogen exchanger (NHE), all of which are associated with tubular factors, and can have some effects on GFR (75). Clues related to this have been proposed in 1994, and a study in diabetic rats indicated that tubular reabsorption may be key to the development of hyperfiltration in DM patients. Besides, K. M Hallow uses a comprehensive mathematical model of the renal vascular system, tubular Na and fluid handling, and systemic blood volume regulation to explore the potential mechanisms of acute and chronic GFR responses to increased tubular Na reabsorption in diabetic patients, which demonstrated that primary tubular hyperabsorption and tubular transport dysregulation determine diabetic glomerular hyperfiltration (81). It was pointed out that tubular cells are able to affect the results and function of glomeruli through alterations in SGLT2, adenosine, ATP, etc. (63). Therefore, tubular function plays an important role in glomerular hyperfiltration, and glomerular hyperfiltration may be secondary to primary tubular injury.

### Tubular Injury Leads to Glomerular Damage

For a long time, DKD has been mainly considered as a diabetic glomerulopathy. However, there is increasing evidence that tubular injury is a key cause of chronic kidney injury (82, 83), which is closely related to the progression of DKD and is superior to glomerular injury as a predictor of DKD progression (84, 85).

Recently, many studies have shown that tubular injury may lead to glomerular disease. Studies have demonstrated that nicotinamide mononucleotide (NMN) released from proximal tubular epithelial cells under high glucose conditions is able to spread to the glomeruli and induces podocyte (PCs) foot process effacement (86). Chunmei Xu has found that tubular Bim is able to mediate tubular-podocyte crosstalk and induces cytoskeletal dysfunction in PCs through activated T cell nuclear factor 2 (NFAT2). Bim is a pro-apoptotic factor involved in the crosstalk between TECs and PCs (87).

Studies have demonstrated that tubular injury not only leads to podocyte disease, but also leads to more extensive glomerular injury. A mouse model of renal injury was established using Six2-Cre-LoxP technique to selectively activate the expression of monkey diphtheria toxin (DT) receptor in tubular epithelial cells. By adjusting the time and dose of DT, a highly selective tubular injury model was created, and this was used to observe the consequences of tubular injury. It was found that after repeated administration of DT, the mice developed maladaptive repair with interstitial capillary loss, fibrosis and glomerulosclerosis. And the degree of glomerulosclerosis is closely related to the degree of tubular injury (88). It demonstrated that tubular injury causes glomerular abnormalities.

The role of renal tubular injury in DKD is getting more and more attention. On the one hand, abnormal elevation of tubular injury markers already occurs before the onset of microalbuminuria in DKD patients, indicating that tubular lesions precede glomerular injury in DKD. On the other hand, renal tubules are more accurate than glomeruli in predicting renal function in DKD, and many studies have also indicated that tubular injury can cause secondary glomerular damage. The study of tubular lesions in DKD provides a new direction to investigate the pathogenesis of DKD.

## Whether Tubular Lipotoxicity Can Be Used as an Indicator for Early Prediction of DKD?

Researchers have made great efforts in understanding the mechanisms of progressive renal decline and developing prognostic tests in DM patients with impaired renal function (89). However, about early renal impairment with DM patients, little is known about the mechanisms, and it lacks early reliable markers. The test of microalbuminuria has been used as the main way to detect early DKD (*93*), but it has recently been challenged. It has been found that some patients with DM may develop DKD even if the urinary albumin levels are within the normal range (90). Therefore, the accuracy and prognostic value of albuminuria in the early stage of DKD are limited. Therefore, there is need to find biomarkers that more accurately predict DKD and its progression in early stage during course.

Whether metabolic disorders are precursors of renal tubules has been proposed. As early as 1982, Moorhead proposed the hypothesis of “lupus nephrotoxicity”, suggesting that abnormal metabolism of lupus may contribute to the progression of renal insufficiency (6). Through metabolic analysis in clinical studies and animal models, it has been shown that alterations in lipid metabolism, TCA cycle, and FAO are the main pathways affecting DKD (91). In addition, the relationship between epithelial transition (EMT) and lipotoxicity in DKD was studied in human proximal tubular cells, and it was found that at 48 h, there was lipid droplet deposit, with more triglycerides, more malonyl-CoA, and lower fatty acid β-oxidation rate, but without morphological change under high glucose conditions. At 96 h, tubular cells became more elongated, less adherent, and lost their apical to basal polarity accompanying with more lipid accumulation (92). These results suggest that FAs deposition has emerged before the induction of EMT phenotype and morphological changes by high glucose. It demonstrated that the progression of lipotoxicity is involved in the development of early DKD before EMT.

A mouse model lacking carnitine acetyltransferase (CRAT) in the tubule was developed to simulate mitochondrial lipid overload. The results showed that mice developed tubular disease, including tubular dilatation, proteinosis, fibrosis, and secondary glomerulosclerosis. When CRAT-null mice were fed a high fat diet, tubular pathological changes occurred 6 months earlier and were more severe than in mice lacking CRAT alone. These results suggest that lipid metabolism disorders may cause changes in kidney functions by affecting the work of mitochondria and promote the progression of DKD (93). Some studies have indicated that mitochondrial dysfunction may be the earliest manifestation of kidney disease, and mitochondrial injury can be used as a marker of tubular injury (94, 95). We have discussed that lipid toxicity can cause mitochondrial dysfunction, which gives us two hints: tubular lipotoxicity may occur before mitochondrial dysfunction and is an earlier event in DKD; tubular lipotoxicity may be an indicator for early prediction of DKD.

Although there are many biomarkers for early detection of DKD in clinical practice, their specificity and sensitivity need to be improved. Our review provides an idea that tubular lipotoxicity may be a major event occurring early in DKD, and has the potential to serve as a marker for early detection of DKD.

## Author Contributions

HW devised the conceptual ideas. HW and SZ drafted the original manuscript and drew the figures. JG edited various versions of the manuscript. All authors read and approved the final manuscript.

## Funding

This work was supported by grants from the National Natural Science Foundation of China (Grant No. 81803823), and Foundation of The Science and Technology Department of Henan Province (Grant No. 182102310540).

## Conflict of Interest

The authors declare that the research was conducted in the absence of any commercial or financial relationships that could be construed as a potential conflict of interest.

## Publisher's Note

All claims expressed in this article are solely those of the authors and do not necessarily represent those of their affiliated organizations, or those of the publisher, the editors and the reviewers. Any product that may be evaluated in this article, or claim that may be made by its manufacturer, is not guaranteed or endorsed by the publisher.

## References

[1] Alicic RZ Rooney MT Tuttle KR. Diabetic kidney disease: challenges, progress, and possibilities. Clin J Am Soc Nephrol. (2017) 12:2032–45. 10.2215/CJN.11491116 28522654 PMC5718284

[2] Cho NH Shaw JE Karuranga S Huang Y Da Rocha Fernandes JD Ohlrogge AW. IDF Diabetes Atlas: Global estimates of diabetes prevalence for 2017 and projections for 2045. Diabetes Res Clin Pr. (2018) 138:271–81. 10.1016/j.diabres.2018.02.023 29496507

[3] Rowley WR Bezold C Arikan Y Byrne E Krohe S. Diabetes 2030: insights from yesterday, today, and future trends. Popul Health Manag. (2017) 20:6–12. 10.1089/pop.2015.0181 27124621 PMC5278808

[4] Tofte N Lindhardt M Adamova K Bakker SJL Beige J Beulens JWJ. Early detection of diabetic kidney disease by urinary proteomics and subsequent intervention with spironolactone to delay progression (PRIORITY): a prospective observational study and embedded randomised placebo-controlled trial. The Lancet Diabetes & Endocrinology. (2020) 8:301–12. 10.1016/S2213-8587(20)30026-7 32135136

[5] Herman-Edelstein M Scherzer P Tobar A Levi M Gafter U. Altered renal lipid metabolism and renal lipid accumulation in human diabetic nephropathy. J Lipid Res. (2014) 55:561–72. 10.1194/jlr.P040501 24371263 PMC3934740

[6] Moorhead JF Chan MK El-Nahas M Varghese Z. Lipid nephrotoxicity in chronic progressive glomerular and tubulo-interstitial disease. Lancet). (1982) 2:1309–11. 10.1016/S0140-6736(82)91513-6 6128601

[7] Lin P Duann P. Dyslipidemia in kidney disorders: perspectives on mitochondria homeostasis and therapeutic opportunities. Front Physiol. (2020) 11:1050. 10.3389/fphys.2020.01050 33013450 PMC7494972

[8] Jang H Noh MR Kim J Padanilam BJ. Defective Mitochondrial Fatty Acid Oxidation and Lipotoxicity in Kidney Diseases. Front Med. (2020) 7:65. 10.3389/fmed.2020.00065 32226789 PMC7080698

[9] Bonventre JV Yang L. Cellular pathophysiology of ischemic acute kidney injury. J Clin Invest. (2011) 121:4210–21. 10.1172/JCI45161 22045571 PMC3204829

[10] Kang HM Ahn SH Choi P Ko Y Han SH Chinga F. Defective fatty acid oxidation in renal tubular epithelial cells has a key role in kidney fibrosis development. Nat Med. (2015) 21:37–46. 10.1038/nm.3762 25419705 PMC4444078

[11] Stadler K Goldberg IJ Susztak K. The evolving understanding of the contribution of lipid metabolism to diabetic kidney disease. Curr Diabetes Rep. (2015) 15:40. 10.1007/s11892-015-0611-8 25957525 PMC4548922

[12] Pepino MY Kuda O Samovski D Abumrad NA. Structure-function of CD36 and importance of fatty acid signal transduction in fat metabolism. Annu Rev Nutr. (2014) 34:281–303. 10.1146/annurev-nutr-071812-161220 24850384 PMC4329921

[13] Storch J Corsico B. The emerging functions and mechanisms of mammalian fatty acid-binding proteins. Annu Rev Nutr. (2008) 28:73–95. 10.1146/annurev.nutr.27.061406.093710 18435590

[14] Susztak K Ciccone E McCue P Sharma K Bottinger EP. Multiple metabolic hits converge on CD36 as novel mediator of tubular epithelial apoptosis in diabetic nephropathy. PLoS Med. (2005) 2:e45. 10.1371/journal.pmed.0020045 15737001 PMC549593

[15] Yang X Okamura DM Lu X Chen Y Moorhead J Varghese Z. CD36 in chronic kidney disease: novel insights and therapeutic opportunities. Nat Rev Nephrol. (2017) 13:769–81. 10.1038/nrneph.2017.126 28919632

[16] Xu H Diolintzi A Storch J. Fatty acid-binding proteins: functional understanding and diagnostic implications. Curr Opin Clin Nutr Metab Care. (2019) 22:407–12. 10.1097/MCO.0000000000000600 31503024 PMC9940447

[17] Tanaka M Furuhashi M Okazaki Y Mita T Fuseya T Ohno K. Ectopic expression of fatty acid-binding protein 4 in the glomerulus is associated with proteinuria and renal dysfunction. Nephron Clin Pract. (2014) 128:345–51. 10.1159/000368412 25592475

[18] Atshaves BP Martin GG Hostetler HA McIntosh AL Kier AB Schroeder F. Liver fatty acid-binding protein and obesity. J Nutr Biochem. (2010) 21:1015–32. 10.1016/j.jnutbio.2010.01.005 20537520 PMC2939181

[19] Houten SM Violante S Ventura FV Wanders RJ. The biochemistry and physiology of mitochondrial fatty acid β -oxidation and its genetic disorders. Ann Rev Physiol. (2016) 78:23–44. 10.1146/annurev-physiol-021115-105045 26474213

[20] Nsiah-Sefaa A McKenzie M. Combined defects in oxidative phosphorylation and fatty acid beta-oxidation in mitochondrial disease. Biosci Rep. (2016) 36:e00313. 10.1042/BSR20150295 26839416 PMC4793296

[21] Katsoulieris E Mabley JG Samai M Sharpe MA Green IC Chatterjee PK. Lipotoxicity in renal proximal tubular cells: relationship between endoplasmic reticulum stress and oxidative stress pathways. Free Radic Biol Med. (2010) 48:1654–62. 10.1016/j.freeradbiomed.2010.03.021 20363316

[22] Chen L Duan Y Wei H Ning H Bi C Zhao Y. Acetyl-CoA carboxylase (ACC) as a therapeutic target for metabolic syndrome and recent developments in ACC1/2 inhibitors. Expert Opin Investig Drugs. (2019) 28:917–30. 10.1080/13543784.2019.1657825 31430206

[23] Thongnak L Pongchaidecha A Lungkaphin A. Renal lipid metabolism and lipotoxicity in diabetes. Am J Med Sci. (2020) 359:84–99. 10.1016/j.amjms.2019.11.004 32039770

[24] Herzig S Shaw RJ. AMPK: guardian of metabolism and mitochondrial homeostasis. Nat Rev Mol Cell Biol. (2018) 19:121–35. 10.1038/nrm.2017.95 28974774 PMC5780224

[25] Russo G Piscitelli P Giandalia A Viazzi F Pontremoli R Fioretto P. Atherogenic dyslipidemia and diabetic nephropathy. J Nephrol. (2020) 33:1001–8. 10.1007/s40620-020-00739-8 32328901

[26] Hou B He P Ma P Yang X Xu C Lam SM. Comprehensive lipidome profiling of the kidney in early-stage diabetic nephropathy. Front Endocrinol. (2020) 11:359. 10.3389/fendo.2020.00359 32655493 PMC7325916

[27] Viji Nair KMS Pradeep Kayampilly JB Jharna Saha HZ. Targeted lipidomic and transcriptomic analysis identifies dysregulated renal ceramide metabolism in a mouse model of diabetic kidney disease. J Proteomics Bioinformat. (2015) 14:002. 10.4172/jpb.S14-002 26778897 PMC4712744

[28] Puchałowicz K Rać ME. The multifunctionality of CD36 in diabetes mellitus and its complications—update in pathogenesis, treatment and monitoring. Cells-Basel. (2020) 9:1877. 10.3390/cells9081877 32796572 PMC7465275

[29] Su W Cao R He YC Guan YF Ruan XZ. Crosstalk of hyperglycemia and dyslipidemia in diabetic kidney disease. Kidney Diseases. (2017) 3:171–80. 10.1159/000479874 29344511 PMC5757547

[30] Park CW Zhang Y Zhang X Wu J Chen L Cha DR. PPARalpha agonist fenofibrate improves diabetic nephropathy in db/db mice. Kidney Int. (2006) 69:1511–7. 10.1038/sj.ki.5000209 16672921

[31] Kim MY Lim JH Youn HH Hong YA Yang KS Park HS. Resveratrol prevents renal lipotoxicity and inhibits mesangial cell glucotoxicity in a manner dependent on the AMPK–SIRT1–PGC1α axis in db/db mice. Diabetologia. (2013) 56:204–17. 10.1007/s00125-012-2747-2 23090186

[32] Hong YA Lim JH Kim MY Kim TW Kim Y Yang KS. Fenofibrate improves renal lipotoxicity through activation of AMPK-PGC-1a in db/dbMice. PLoS ONE. (2014) 9:e96147. 10.1371/journal.pone.0096147 24801481 PMC4011795

[33] Koh ES Lim JH Kim MY Chung S Shin SJ Choi BS. Anthocyanin-rich Seoritae extract ameliorates renal lipotoxicity via activation of AMP-activated protein kinase in diabetic mice. J Transl Med. (2015) 13:203. 10.1186/s12967-015-0563-4 26116070 PMC4482313

[34] Cusi K. Role of obesity and lipotoxicity in the development of nonalcoholic steatohepatitis: pathophysiology and clinical implications. Gastroenterology. (2012) 142:711–25. 10.1053/j.gastro.2012.02.003 22326434

[35] Opazo-Ríos L Mas S Marín-Royo G Mezzano S Gómez-Guerrero C Moreno JA. Lipotoxicity and diabetic nephropathy: novel mechanistic insights and therapeutic opportunities. Int J Mol Sci. (2020) 21:2632. 10.3390/ijms21072632 32290082 PMC7177360

[36] Murea M Freedman BI Parks JS Antinozzi PA Elbein SC Ma L. Lipotoxicity in diabetic nephropathy: the potential role of fatty acid oxidation. Clin J Am Soc Nephrol. (2010) 5:2373–9. 10.2215/CJN.08160910 21051750

[37] Atageldiyeva KK Nemr R Echtay A Racoubian E Sarray S Almawi WY. Apolipoprotein E genetic polymorphism influence the susceptibility to nephropathy in type 2 diabetes patients. Gene. (2019) 715:144011. 10.1016/j.gene.2019.144011 31357022

[38] Jankovic M Novakovic I Nikolic D Mitrovic MJ Brankovic S Petronic I. Genetic and Epigenomic Modifiers of Diabetic Neuropathy. Int J Mol Sci. (2021) 22:4887. 10.3390/ijms22094887 34063061 PMC8124699

[39] Izquierdo-Lahuerta A Martínez-García C Medina-Gómez G. Lipotoxicity as a trigger factor of renal disease. J Nephrol. (2016) 29:603–10. 10.1007/s40620-016-0278-5 26956132

[40] Nishi H Higashihara T Inagi R. Lipotoxicity in kidney, heart, and skeletal muscle dysfunction. Nutrients. (2019) 11:1664. 10.3390/nu11071664 31330812 PMC6682887

[41] Schelling JR. Tubular atrophy in the pathogenesis of chronic kidney disease progression. Pediatr Nephrol. (2016) 31:693–706. 10.1007/s00467-015-3169-4 26208584 PMC4726480

[42] Ge M Fontanesi F Merscher S Fornoni A. The vicious cycle of renal lipotoxicity and mitochondrial dysfunction. Front Physiol. (2020) 11:732. 10.3389/fphys.2020.00732 32733268 PMC7358947

[43] Jao TM Nangaku M Wu CH Sugahara M Saito H Maekawa H. ATF6? downregulation of PPARa promotes lipotoxicity-induced tubulointerstitial fibrosis. Kidney Int. (2019) 95:577–89. 10.1016/j.kint.2018.09.023 30639234

[44] Iwai T Kume S Chin-Kanasaki M Kuwagata S Araki H Takeda N. Stearoyl-CoA desaturase-1 protects cells against lipotoxicity-mediated apoptosis in proximal tubular cells. Int J Mol Sci. (2016) 17:1868. 10.3390/ijms17111868 27834856 PMC5133868

[45] Erkan E Devarajan P Schwartz GJ. Mitochondria are the major targets in albumin-induced apoptosis in proximal tubule cells. J Am Soc Nephrol. (2007) 18:1199–208. 10.1681/ASN.2006040407 17360944

[46] Russo LM Sandoval RM McKee M Osicka TM Collins AB Brown D. The normal kidney filters nephrotic levels of albumin retrieved by proximal tubule cells: retrieval is disrupted in nephrotic states. Kidney Int. (2007) 71:504–13. 10.1038/sj.ki.5002041 17228368

[47] Sakashita M Tanaka T Inagi R. Metabolic changes and oxidative stress in diabetic kidney disease. Antioxidants. (2021) 10:1143. 10.3390/antiox10071143 34356375 PMC8301131

[48] Mitrofanova A Burke G Merscher S Fornoni A. New insights into renal lipid dysmetabolism in diabetic kidney disease. World J Diabetes. (2021) 12:524–40. 10.4239/wjd.v12.i5.524 33995842 PMC8107981

[49] Chen Y Dai Y Song K Huang Y Zhang L Zhang C. Pre-emptive pharmacological inhibition of fatty acid-binding protein 4 attenuates kidney fibrosis by reprogramming tubular lipid metabolism. Cell Death Dis. (2021) 12:572. 10.1038/s41419-021-03850-1 34083513 PMC8175732

[50] Escasany E Izquierdo-Lahuerta A Medina-Gomez G. Underlying mechanisms of renal lipotoxicity in obesity. Nephron. (2019) 143:28–32. 10.1159/000494694 30625473

[51] Tanaka Y Kume S Araki H Nakazawa J Chin-Kanasaki M Araki S. 1-Methylnicotinamide ameliorates lipotoxicity-induced oxidative stress and cell death in kidney proximal tubular cells. Free Radic Biol Med. (2015) 89:831–41. 10.1016/j.freeradbiomed.2015.10.414 26482866

[52] Chen X Han Y Gao P Yang M Xiao L Xiong X. Disulfide-bond A oxidoreductase-like protein protects against ectopic fat deposition and lipid-related kidney damage in diabetic nephropathy. Kidney Int. (2019) 95:880–95. 10.1016/j.kint.2018.10.038 30791996

[53] Yang W Luo Y Yang S Zeng M Zhang S Liu J. Ectopic lipid accumulation: potential role in tubular injury and inflammation in diabetic kidney disease. Clin Sci (Lond). (2018) 132:2407–22. 10.1042/CS20180702 30348828

[54] Pérez-Morales RE Del Pino MD Valdivielso JM Ortiz A Mora-Fernández C Navarro-González JF. Inflammation in Diabetic Kidney Disease. Nephron. (2015) 143:12–6. 10.1159/000493278 30273931

[55] Che R Yuan Y Huang S Zhang A. Mitochondrial dysfunction in the pathophysiology of renal diseases. Am J Physiol Renal Physiol. (2014) 306:F367–78. 10.1152/ajprenal.00571.2013 24305473

[56] Coughlan MT Nguyen T Penfold SA Higgins GC Thallas-Bonke V Tan SM. Mapping time-course mitochondrial adaptations in the kidney in experimental diabetes. Clin Sci. (2016) 130:711–20. 10.1042/CS20150838 26831938

[57] Pisoschi AM Pop A. The role of antioxidants in the chemistry of oxidative stress: A review. Eur J Med Chem. (2015) 97:55–74. 10.1016/j.ejmech.2015.04.040 25942353

[58] Hosohata K. Role of oxidative stress in drug-induced kidney injury. Int J Mol Sci. (2016) 17:1826. 10.3390/ijms17111826 27809280 PMC5133827

[59] Zhao HL Sui Y Guan J He L Zhu X Fan RR. Fat redistribution and adipocyte transformation in uninephrectomized rats. Kidney Int. (2008) 74:467–77. 10.1038/ki.2008.195 18496513

[60] Li C Lin Y Luo R Chen S Wang F Zheng P. Intrarenal renin-angiotensin system mediates fatty acid-induced ER stress in the kidney. Am J Physiol Renal Physiol. (2016) 310:F351–63. 10.1152/ajprenal.00223.2015 26672616 PMC4971807

[61] Zhou H Liu R. ER stress and hepatic lipid metabolism. Front Genet. (2014) 5:112. 10.3389/fgene.2014.00112 24847353 PMC4023072

[62] Yang L Guan G Lei L Liu J Cao L Wang X. Oxidative and endoplasmic reticulum stresses are involved in palmitic acid-induced H9c2 cell apoptosis. Biosci Rep. (2019) 39:BSR20190225. 10.1042/BSR20190225 31064816 PMC6527925

[63] Chen SJ Lv LL Liu BC Tang RN. Crosstalk between tubular epithelial cells and glomerular endothelial cells in diabetic kidney disease. Cell Proliferat. (2020) 53:e12763. 10.1111/cpr.12763 31925859 PMC7106959

[64] Persson F Rossing P. Diagnosis of diabetic kidney disease: state of the art and future perspective. Kidney Int Suppl. (2011) 8:2–7. 10.1016/j.kisu.2017.10.003 30675433 PMC6336222

[65] Tonneijck L Muskiet MH Smits MM van Bommel EJ Heerspink HJ van Raalte DH. Glomerular hyperfiltration in diabetes: mechanisms, clinical significance, and treatment. J Am Soc Nephrol. (2017) 28:1023–39. 10.1681/ASN.2016060666 28143897 PMC5373460

[66] Magee GM Bilous RW Cardwell CR Hunter SJ Kee F Fogarty DG. Is hyperfiltration associated with the future risk of developing diabetic nephropathy? A meta-analysis Diabetologia. (2009) 52:691–7. 10.1007/s00125-009-1268-0 19198800

[67] Goncalves-Dias C Morello J Correia MJ Coelho NR Antunes A Macedo MP. Mercapturate pathway in the tubulocentric perspective of diabetic kidney disease. Nephron. (2019) 143:17–23. 10.1159/000494390 30625494

[68] Hafez MH El-Mougy FA Makar SH Abd ESS. Detection of an earlier tubulopathy in diabetic nephropathy among children with normoalbuminuria. Iran J Kidney Dis. (2015) 9:126–31. 25851291

[69] Gilbert RE. Proximal Tubulopathy: Prime Mover and Key Therapeutic target in diabetic kidney disease. Diabetes. (2017) 66:791–800. 10.2337/db16-0796 28325740

[70] Zeni L Norden AGW Cancarini G Unwin RJ. A more tubulocentric view of diabetic kidney disease. J Nephrol. (2017) 30:701–17. 10.1007/s40620-017-0423-9 28840540 PMC5698396

[71] Thethi TK Batuman V. Challenging the conventional wisdom on diabetic nephropathy: Is microalbuminuria the earliest event? J Diabetes Complications. (2019) 33:191–2. 10.1016/j.jdiacomp.2018.12.006 30651179

[72] Wagner MC Campos-Bilderback SB Chowdhury M Flores B Lai X Myslinski J. Proximal tubules have the capacity to regulate uptake of albumin. J Am Soc Nephrol. (2016) 27:482–94. 10.1681/ASN.2014111107 26054544 PMC4731114

[73] Comper WD. Albuminuria is controlled primarily by proximal tubules. Nat Rev Nephrol. (2014) 10:180. 10.1038/nrneph.2013.58-c1 24468765

[74] Russo LM Srivatsan S Seaman M Suleiman H Shaw AS Comper WD. Albuminuria associated with CD2AP knockout mice is primarily due to dysfunction of the renal degradation pathway processing of filtered albumin. FEBS Lett. (2013) 587:3738–41. 10.1016/j.febslet.2013.09.045 24140342

[75] Yu Y Jin H Holder D Ozer JS Villarreal S Shughrue P. Urinary biomarkers trefoil factor 3 and albumin enable early detection of kidney tubular injury. Nat Biotechnol. (2010) 28:470–7. 10.1038/nbt.1624 20458317

[76] Gibb DM Tomlinson PA Dalton NR Turner C Shah V Barratt TM. Renal tubular proteinuria and microalbuminuria in diabetic patients. Arch Dis Child. (1989) 64:129–34. 10.1136/adc.64.1.129 2923463 PMC1791792

[77] Vallon V Thomson SC. The tubular hypothesis of nephron filtration and diabetic kidney disease. Nature reviews Nephrology. (2020) 16:317–36. 10.1038/s41581-020-0256-y 32152499 PMC7242158

[78] Vallon V Thomson SC. Renal function in diabetic disease models: the tubular system in the pathophysiology of the diabetic kidney. Annu Rev Physiol. (2012) 74:351–75. 10.1146/annurev-physiol-020911-153333 22335797 PMC3807782

[79] Fu W Li B Wang S Chen M Deng R Ye C. Changes of the tubular markers in type 2 diabetes mellitus with glomerular hyperfiltration. Diabetes Res Clin Pr. (2012) 95:105–9. 10.1016/j.diabres.2011.09.031 22015481

[80] Cherney DZ Perkins BA Soleymanlou N Maione M Lai V Lee A. Renal hemodynamic effect of sodium-glucose cotransporter 2 inhibition in patients with type 1 diabetes mellitus. Circulation. (2014) 129:587–97. 10.1161/CIRCULATIONAHA.113.005081 24334175

[81] Hallow KM Gebremichael Y Helmlinger G Vallon V. Primary proximal tubule hyperreabsorption and impaired tubular transport counterregulation determine glomerular hyperfiltration in diabetes: a modeling analysis. Am J Physiol-Renal. (2017) 312:F819–35. 10.1152/ajprenal.00497.2016 28148531 PMC5451553

[82] Xu L Li X Zhang F Wu L Dong Z Zhang D. drives the progression of AKI to CKD through HIPK2 overexpression. Theranostics. (2019) 9:2712–26. 10.7150/thno.31424 31131063 PMC6526000

[83] Pan T Jia P Chen N Fang Y Liang Y Guo M. Delayed remote ischemic preconditioning confersrenoprotection against septic acute kidney injury via exosomal miR-21. Theranostics. (2019) 9:405–23. 10.7150/thno.29832 30809283 PMC6376188

[84] Fu Y Wang C Zhang D Xin Y Li J Zhang Y. Increased TRPC6 expression is associated with tubular epithelial cell proliferation and inflammation in diabetic nephropathy. Mol Immunol. (2018) 94:75–81. 10.1016/j.molimm.2017.12.014 29288897

[85] Satirapoj B Aramsaowapak K Tangwonglert T Supasyndh O. Novel tubular biomarkers predict renal progression in type 2 diabetes mellitus: a prospective cohort study. J Diabetes Res. (2016) 2016:1–9. 10.1155/2016/3102962 27672664 PMC5031837

[86] Hasegawa K Wakino S Simic P Sakamaki Y Minakuchi H Fujimura K. Renal tubular Sirt1 attenuates diabetic albuminuria by epigenetically suppressing Claudin-1 overexpression in podocytes. Nat Med. (2013) 19:1496–504. 10.1038/nm.3363 24141423 PMC4041199

[87] Xu C Zhou X Xie T Zhou Y Zhang Q Jiang S. Renal tubular Bim mediates the tubule-podocyte crosstalk via NFAT2 to induce podocyte cytoskeletal dysfunction. Theranostics. (2020) 10:6806–24. 10.7150/thno.43145 32550905 PMC7295056

[88] Grgic I Campanholle G Bijol V Wang C Sabbisetti VS Ichimura T. Targeted proximal tubule injury triggers interstitial fibrosis and glomerulosclerosis. Kidney Int. (2012) 82:172–83. 10.1038/ki.2012.20 22437410 PMC3480325

[89] Nowak N Skupien J Smiles AM Yamanouchi M Niewczas MA Galecki AT. Markers of early progressive renal decline in type 2 diabetes suggest different implications for etiological studies and prognostic tests development. Kidney Int. (2018) 93:1198–206. 10.1016/j.kint.2017.11.024 29398132 PMC5911430

[90] Jiang W Wang J Shen X Lu W Wang Y Li W. Establishment and validation of a risk prediction model for early diabetic kidney disease based on a systematic review and meta-analysis of 20 cohorts. Diabetes Care. (2020) 33:925–33. 10.2337/dc19-1897 32198286

[91] Darshi M Van Espen B Sharma K. Metabolomics in diabetic kidney disease: unraveling the biochemistry of a silent killer. Am J Nephrol. (2016) 44:92–103. 10.1159/000447954 27410520 PMC6581452

[92] Xu Y Huang J Xin W Chen L Zhao X Lv Z. Lipid accumulation is ahead of epithelial-to-mesenchymal transition and therapeutic intervention by acetyl-CoA carboxylase 2 silence in diabetic nephropathy. Metabolism. (2014) 63:716–26. 10.1016/j.metabol.2014.02.010 24650564

[93] Kruger C Nguyen TT Breaux C Guillory A Mangelli M Fridianto KT. Proximal tubular cell-specific ablation of carnitine acetyltransferase causes tubular disease and secondary glomerulosclerosis. Diabetes. (2019) 68:819–31. 10.2337/db18-0090 30728184 PMC6425873

[94] Jiang H Shao X Jia S Qu L Weng C Shen X. The mitochondria-targeted metabolic tubular injury in diabetic kidney disease. Cell Physiol Biochem. (2019) 52:156–71. 10.33594/000000011 30816665

[95] Wei PZ Szeto CC. Mitochondrial dysfunction in diabetic kidney disease. Clin Chim Acta. (2019) 496:108–16. 10.1016/j.cca.2019.07.005 31276635

